# Achieving thermally stable nanoparticles in chemically complex alloys via controllable sluggish lattice diffusion

**DOI:** 10.1038/s41467-022-32620-6

**Published:** 2022-08-18

**Authors:** Bo Xiao, Junhua Luan, Shijun Zhao, Lijun Zhang, Shiyao Chen, Yilu Zhao, Lianyong Xu, C. T. Liu, Ji-Jung Kai, Tao Yang

**Affiliations:** 1 Department of Materials Science and Engineering, City University of Hong Kong, Hong Kong, China; 2 Department of Mechanical Engineering, City University of Hong Kong, Hong Kong, China; 3 Hong Kong Institute for Advanced Study, City University of Hong Kong, Hong Kong, China; 4 State Key Laboratory of Powder Metallurgy, Central South University, Changsha, 410083 China; 5 School of Materials Science and Engineering, Harbin Institute of Technology (Shenzhen), Shenzhen, 518055 China; 6 School of Materials Science and Engineering, Tianjin University, Tianjin, 300350 China

**Keywords:** Structural materials, Nanoscale materials

## Abstract

Nanoparticle strengthening provides a crucial basis for developing high-performance structural materials with potentially superb mechanical properties for structural applications. However, the general wisdom often fails to work well due to the poor thermal stability of nanoparticles, and the rapid coarsening of these particles will lead to the accelerated failures of these materials especially at elevated temperatures. Here, we demonstrate a strategy to achieve ultra-stable nanoparticles at 800~1000 °C in a Ni_59.9-*x*_Co_*x*_Fe_13_Cr_15_Al_6_Ti_6_B_0.1_ (at.%) chemically complex alloy, resulting from the controllable sluggish lattice diffusion (SLD) effect. Our diffusion kinetic simulations reveal that the Co element leads to a significant reduction in the interdiffusion coefficients of all the main elements, especially for the Al element, with a maximum of up to 5 orders of magnitude. Utilizing first-principles calculations, we further unveil the incompressibility of Al induced by the increased concentration of Co plays a critical role in controlling the SLD effect. These findings are useful for providing advances in the design of novel structural alloys with extraordinary property-microstructure stability combinations for structural applications.

## Abstract (web summary)

Nanoparticle strengthening provides a crucial basis for developing high-performance materials, which often fails to work due to poor thermal stability. Here, the authors achieve thermally stable nanoparticles at 800~1000 °C in chemically complex alloys via controllable sluggish lattice diffusion.

## Introduction

Materials containing well-stabilized nanostructures have rendered salient advantages in seeking unique property combinations, including both structural and functional, holding a great promise for achieving improved energy efficiency and carbon neutrality^1–5^. Of particular interest, “nanoparticle strengthening,” as a powerful strategy, has been widely applied for the innovation of high-strength materials like advanced Al alloys^6,7^, steels^8–11^, and superalloys^12–14^, all of which play crucial roles in various technologic and industrial fields, such as aerospace, automotive, and nuclear engineering. Unfortunately, these second-phase fine particles at the nanoscale are inevitably prone to rapid coarsening, which dramatically decreases the load-carrying capacity of host materials and consequently leads to catastrophic failures^15–18^. Although numerous efforts have been made^19,20^, such an undesired coarsening behavior persists as the Achilles’ heel for many structural alloys, especially for those serving at elevated temperatures. Notably, the recent discovery of chemically complex alloys (CCAs) has been demonstrated as a new paradigm for developing novel structural materials with unique physical and mechanical properties^21–29^. In particular, the so-called sluggish lattice diffusion (SLD) effect^30–33^ potentially endows several CCAs with remarkable thermal stability^34–40^. Until now, however, due to the lack of quantitative understanding, the underlying mechanism of SLD effect has not been well elucidated and its atomistic origin still remains mysterious at the present time. This, frustratingly, makes the achieving of ultra-stable nanostructures (USNSs) in CCAs to be uncontrollable.

In the present study, through a combination of various complementary experimental techniques and theoretical simulations, we find the key to effectively stabilizing the second-phase nanoparticles in a chemically complex NiCoFeCrAlTiB high-entropy metallic system. More specifically, we reveal that tailoring the concentration of the Co element can controllably govern the SLD effect in a quantitative manner, which enables us to substantially prevent the nanoparticles from rapid coarsening at high temperatures up to 1000 °C. These results can pave the way to develop an efficient design of high-performance alloys with both good mechanical and thermal properties for high-temperature structural applications.

## Results and discussion

**Fig. 1:**
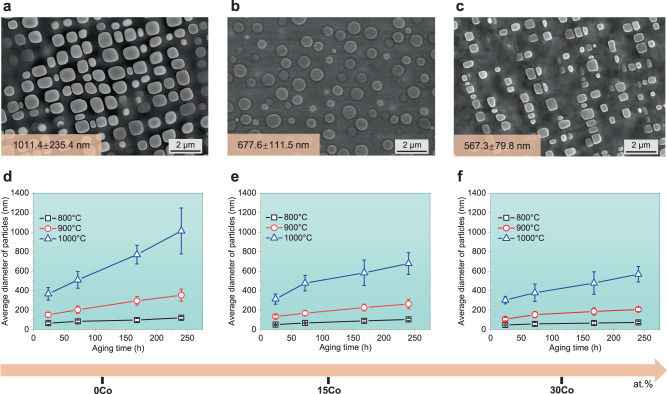
Enhanced thermal stability in the studied CCAs with the addition of Co element (0, 15, and 30 at.%). **a**–**c** Typical SEM micrographs of nanoparticles in the three CCAs aged at 1000 °C for 240 h, and their average diameters are separately evaluated to be 1011.4 ± 235.4 nm, 677.6 ± 111.5 nm, and 567.3 ± 79.8 nm. **d**–**f** The evolution of the average size of nanoparticles in the three CCAs aged at 800, 900, and 1000 °C for different durations (24 h, 72 h, 168 h, and 240 h).

To explore the significant effect of the Co element on the sluggish diffusion kinetics in CCAs, three experimental alloys, Ni_59.9-*x*_Co_*x*_Fe_13_Cr_15_Al_6_Ti_6_B_0.1_ (*x* = 0, 15, and 30 at.%, denoted as the 0Co, 15Co, and 30Co CCAs), were cast via arc melting, and subsequently, followed by thermally-aging processing (see details in “**Methods**”). As displayed in Fig. 1a–c, the average sizes of nanoparticles for the three CCAs with 0Co, 15Co, and 30Co are respectively evaluated to be 1011.4 ± 235.4 nm (Fig. 1a), 677.6 ± 111.5 nm (Fig. 1b), and 567.3 ± 79.8 nm (Fig. 1c) aged at 1000 °C for 240 h. We also showcase the typical scanning electron microscopy (SEM) micrographs of the 0Co, 15Co, and 30Co CCAs at 800, 900, and 1000 °C for different durations (24 h, 72 h, 168 h, and 240 h), see Supplementary Figs. 1–3. We further quantitatively evaluate the average size evolutions of nanoparticles in the three CCAs with the variation of aging time at different temperatures (see details in “**Methods**”), as provided in Fig. 1d–f. Our experiments indicate that the increased concentrations of Co element can substantially reduce the average particle size and further improve the thermal stability of these nanoparticles.

**Fig. 2:**
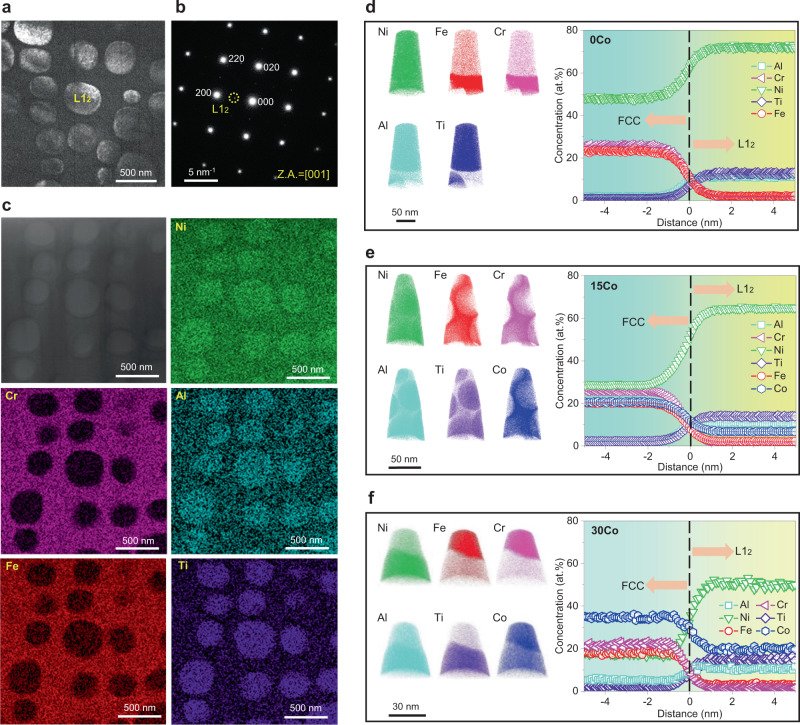
Structure characterization and chemical composition of nanoparticles. **a** A typical dark-field TEM micrograph of nanoparticles in the 0Co CCA aged at 1000 °C for 24 h. **b** Nanoparticles are identified as L1_2_ precipitates with the aid of SAED pattern (Z.A. denotes the zone axis). **c** The scanning TEM-EDS maps reveal that L1_2_ precipitates are rich in Ni, Al, and Ti. **d**–**f** 3D-APT reconstruction of the tip and the corresponding compositional profile across the FCC/L1_2_ interface from the 0Co, 15Co, and 30Co samples aged at 800 °C for 24 h.

We further characterize the crystal structure and chemical composition of nanoparticles by using transmission electron microscopy (TEM) and atom probe tomography (APT) techniques. Combining the selected area electron diffraction (SAED) pattern (Fig. 2b) and chemical composition analysis (Fig. 2c), the nanoparticles and matrix, as seen in Fig. 2a, possess L1_2_ and face-centered cubic (FCC) structures, respectively. Furthermore, we also characterize the elemental partitioning in the two phases (FCC + L1_2_) for the 0Co, 15Co, and 30Co CCAs aged at 800 °C for 24 h, as displayed in Fig. 2d–f. Apparently, we can see that Ti, Al, and Ni elements primarily partition to the L1_2_ precipitates whereas Fe and Cr are largely depleted from the precipitates for the 0Co CCA (Fig. 2d), in agreement with our TEM energy dispersive spectroscopy (EDS) result (Fig. 2c). In contrast, Co has a strong partition into the FCC matrix in the 15Co and 30Co CCAs (Fig. 2e, f). Similarly, the elemental partitioning behaviors in the FCC matrix and L1_2_ precipitates for the 0Co and 15Co CCAs aged at 900 °C for 24 h are also evaluated, as seen in Supplementary Figs. 4 and 5. Based on the APT and Scanning TEM-EDS results, we summarize the chemical composition and partitioning of L1_2_ precipitates and the FCC matrix of the 0Co and 15Co alloys at 800, 900, and 1000 °C for 24 h, as shown in Supplementary Table. 1, which provides basic data for our subsequent diffusion kinetic simulations. Notably, the refractory elements such as rhenium (Re) are generally included in metallic alloys to reduce the solute diffusion kinetics via solute segregation at phase interfaces^39^, whereas in the current study, no elemental segregation at the L1_2_/FCC interface is detected (Fig. 2d–f, Supplementary Figs. 4 and 5) and thus a more in-depth understanding for the Co element-governed sluggish diffusion mechanism is required.

**Fig. 3:**
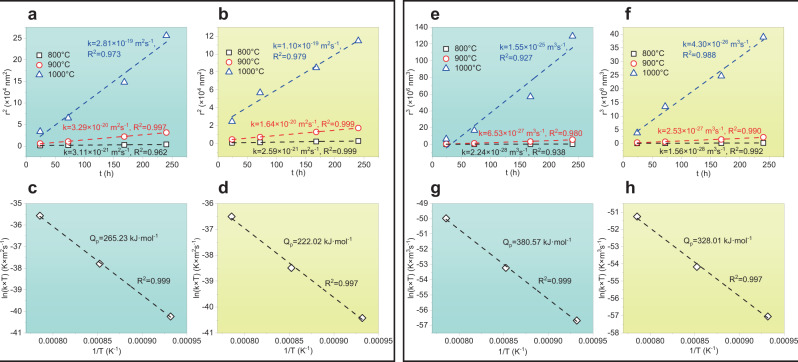
Coarsening kinetics and associated mechanisms of L1_2_ precipitates in the 0Co and 15Co CCAs. **a**, **b** Plots of *r*^2^ vs. *t* of L1_2_ precipitates in the 0Co and 15Co CCAs aged at 800, 900, and 1000 °C. **c**, **d** Arrhenius plot of the coarsening rate constant (ln(k × T)) as a function of the reciprocal aging temperature (1/T) determines the activation energy (*Q*) for the 0Co and 15Co CCAs based on the TIDC model. **e**, **f** Plots of *r*^3^ vs. *t* of L1_2_ precipitates in the 0Co and 15Co CCAs aged at 800, 900, and 1000 °C. **g**, **h** Arrhenius plot of the coarsening rate constant (ln(k × T)) as a function of the reciprocal aging temperature (1/T) gives the activation energy (*Q*) for the 0Co and 15Co CCAs based on the LSW model.

We next quantitatively discuss the coarsening kinetics and associated mechanisms of L1_2_ precipitates in the studied CCAs. It is well known that the coarsening behaviors of the L1_2_ precipitates can be described by the classical Ostwald ripening process^41,42^. Regarding our studied CCAs, the coarsening kinetics is controlled by two factors: solute diffusion through the FCC matrix and the FCC/L1_2_ interface. Such a time-dependent coarsening process can be described by the power-law expression^43,44^,

1 $${r}^{{{{{{\rm{n}}}}}}}(t)-{r}^{{{{{{\rm{n}}}}}}}({t}_{0})=k(t-{t}_{0})$$

where *r*(*t*) is the average radius of the precipitate at the aging time of *t*, *k* is the coarsening rate constant. *n* = 3 and 2 represent that the coarsening kinetics are generally governed by the volume-diffusion mechanism (LSW) and by the trans-interface diffusion controlled (TIDC) mechanism^37,45^, respectively. The linear fit to the data points of *r*^3^ vs. *t* and *r*^2^ vs. *t* during thermal aging and corresponding activation energies are displayed in Fig. 3. Considering the coarsening kinetics and calculated activation energy (more details are provided in Supplementary Note 1 and Supplementary Fig. 6), our results suggest that the coarsening behavior of L1_2_ precipitates in the 0Co CCA is controlled by the TIDC mechanism, but that in the 15Co CCA is governed by the LSW mechanism. Notably, the coarsening of L1_2_ precipitates in the 30Co CCA is also dominated by the LSW mechanism^35^. Therefore, our coarsening kinetic analyses reveal that the Co element induces the transition of the coarsening mechanisms of L1_2_ precipitates in the studied CCAs.

**Fig. 4:**
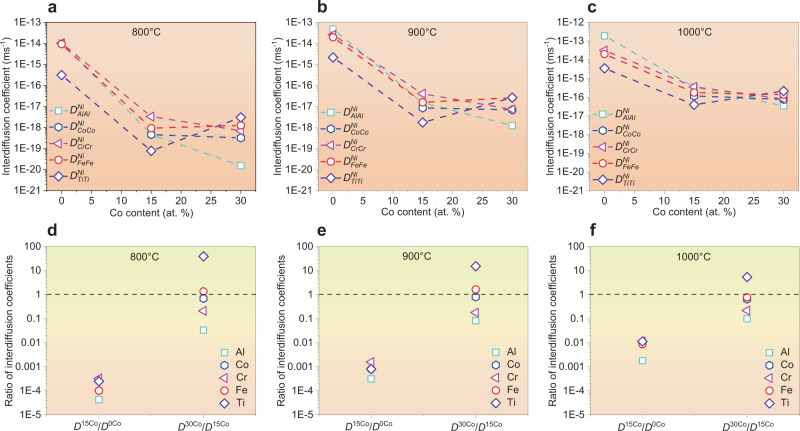
Calculated main interdiffusion coefficients of Al, Co, Cr, Fe, and Ti elements in the FCC matrices (with Ni as the solvent) in the Ni_59.9-*x*_Co_*x*_Fe_13_Cr_15_Al_6_Ti_6_B_0.1_ (at.%) CCA system. **a**–**c** 800 °C, 900 °C, and 1000 °C. **d**–**f** The variations of the ratio of interdiffusion coefficients with increasing the Co content at 800 °C, 900 °C, and 1000 °C.

It has been recognized that the coarsening rate of a particle is mainly controlled by the rate-limiting step^46^. From our quantitative experimental analyses, as seen in Fig. 3, the L1_2_/FCC interface reaction is the rate-limiting step in the 0Co CCA, while for the 15Co and 30Co CCAs, the LSW mechanism is the rate-limiting step. So far it is still challenging for a calculation on the solute diffusion kinetics at the FCC/L1_2_ interface. To unveil the transition of coarsening mechanism of L1_2_ precipitates induced by the Co element, we focus on the essential role of the Co element in reducing diffusion kinetics through the FCC matrix in the studied systems. Here, utilizing a combination of HitDIC software (High-throughput Determination of Interdiffusion Coefficients, https://hitdic.com/) and NiCoFeCrAlTi diffusion multiples^47^, we have calculated the variation of interdiffusion coefficients of Al, Co, Cr, Fe, and Ti elements in the FCC matrices in the studied CCAs with different levels of the Co content (0, 15, and 30Co at.%). As demonstrated in Fig. 4, all the main interdiffusion coefficients decrease with increasing the Co content. Surprisingly, the interdiffusion coefficient of Al element decreases from 1.06 × 10^−14 ^ms^−1^ to 1.53 × 10^−20 ^ms^−1^, up to 5 orders of magnitude at 800 °C as the Co content increases from 0 to 30 at.% (Fig. 4d). Meanwhile, it also reveals that the Co element leads to the greatest reduction in the interdiffusion coefficients of the Al element when compared to other elements like Co, Cr, Fe, and Ti (Fig. 4d–f). The simulated interdiffusion coefficients of Al, Co, Cr, Fe, and Ti elements in the FCC matrices (with Ni as the solvent) in the three CCAs at different temperatures (800, 900, and 1000 °C) are provided in Supplementary Table 3. As a result, the Co element-induced reduction in the interdiffusion coefficients among all the main elements is responsible for the transition of coarsening mechanisms in the studied CCAs.

**Fig. 5:**
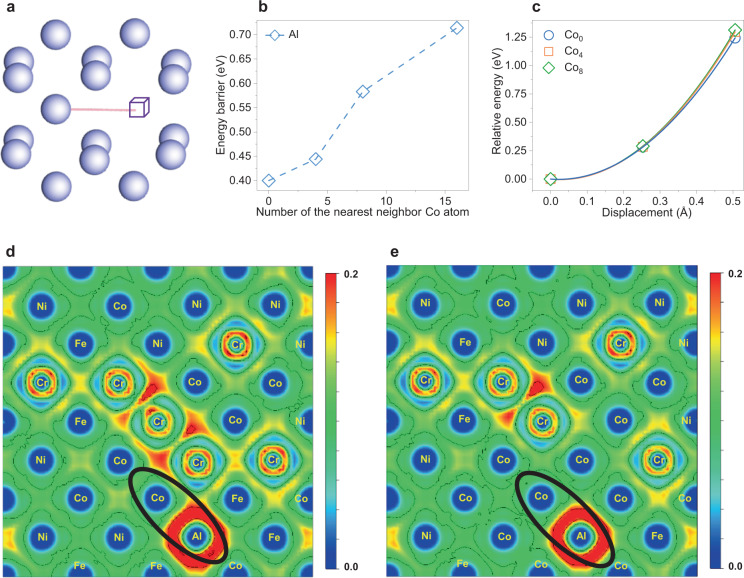
The origin of the nearest neighbor Co atoms on Al diffusion by first-principles calculations. **a** The migration path of the exchange between a lattice atom and a nearby vacancy. **b** Migration barrier of an Al atom with the increase of the number of the nearest neighbor Co atoms (0, 4, 8, and 16). **c** Relative energy changes when an Al is moved towards a Co atom under different Co environments. **d**, **e** ELF plot in a [100] plane corresponding to the case of **c** at different numbers of the nearest neighbor Co atoms. Note that ELF = 0 and 1 represent the completely delocalized and perfect localization of electrons, respectively. Color scale of the ELF value difference ranges from 0 to 0.2.

Based on our diffusion kinetic calculations (Fig. 4), we determine that the Co element greatly affects the interdiffusion behavior of the Al element, which probably plays an unexpected role in the controlling SLD effect. Here, using first-principles calculations based on density-functional theory (DFT), we further elucidate the critical origin of the reduction in the interdiffusion coefficients of the Al element-induced by the neighboring Co element. As displayed in Fig. 5b, with the increase of the number of the nearest neighbor Co atoms, the energy barrier for the Al atom increases significantly, suggesting that a higher Co concentration will suppress the diffusion of the Al atom. To further probe the effects of the Co atom on the diffusion of Al atom, we have studied the compression properties of an Al atom towards the Co atom. As shown in Fig. 5c, the relative energy increases with the shortage of bond length of Co-Al. Notably, the energy change increases with increasing the number of the nearest neighbor Co atoms. The corresponding electron localized function (ELF) plots are provided in Fig. 5d, e. The distribution of the ELF can be used to analyze interatomic interactions inside the crystal structure. Specifically, ELF = 0 and 1 represent the completely delocalized and perfect localization of electrons, respectively^48^. For the CCAs considered, the ELF values are <0.5, indicating delocalized electron states^49^. With increasing the number of the nearest neighbor Co atoms, we find the ELF values of the system increase, indicative of the localization of electrons induced by Co atoms, as shown in Fig. 5d, e, Supplementary Table 4. Inspection of the surroundings of Al atoms suggests that most electrons are localized around Al atoms, and the ELF values are quite low in the Al-Co bond regions. There is no electron partition between Al-Co atoms, making the bonding interactions stiff. As a result, the high Co concentrations surrounding Al atom lead to less compressibility compared to the random distribution of Ni/Co/Fe/Cr elements. It has been well established that the migration barriers are sensitive to the compressibility of elements^50^, since the migration atom should undergo a considerable compression force in the saddle structure (the atom is exactly located in the middle of the path). Therefore, the incompressibility of Al induced by the high concentration of Co is responsible for the high migration barriers and low diffusivity of Al, which offers an in-depth understanding of achieving USNSs in the CCAs by adding appropriate Co elements.

It is worth noting that the L2_1_-type Heusler phase starts to form at grain boundaries when the Co content increases up to 30 at.%, as shown in Supplementary Fig. 7. The Heusler phase often causes increased local stress concentration and premature failures of the alloys as it is extremely brittle and incoherent with the FCC matrix^35,51^. To further unravel the effect of a more Co solute on the phase stability of this studied system, we also fabricate Co_59.9_Fe_13_Cr_15_Al_6_Ti_6_B_0.1_ CCA (referred to as 60Co CCA). Typical SEM images (Supplementary Fig. 8) present the precipitate morphologies and distributions of the 60Co CCA aged at 1000 °C for 24 and 72 h, apparently different from those of the other three CCAs (Supplementary Figs. 1–3). The precipitates are identified as the B2 phase with the aid of TEM and SAED pattern (Supplementary Fig. 9). Tensile curves (Supplementary Fig. 10) further demonstrate that the 60Co CCA shows an inferior tensile strength and ductility when compared to the 30Co CCA. Based on the microstructural observations, we demonstrate the evolution of phase structures in our CCA system with increasing the Co content, see Supplementary Fig. 11. Combining the mechanical data and the phase structures, it implies that an appropriate range of the Co content (15~30 at.%) in such a CCA system can greatly improve the microstructure stability at elevated temperatures.

In conclusion, we propose a controllable SLD strategy to achieve USNSs in the CCA systems at elevated temperatures. Both experimental and simulated results demonstrate that tailoring the concentration of Co element significantly improves the thermal stability of nanoparticles by decreasing interdiffusion coefficients of other elements, especially for the Al element. Our theoretical calculations show that the incompressibility of Al, induced by a high concentration of Co, leads to the low diffusivity of the Al atom, which is the critical origin of such Co element-governed SLD effect in CCAs. The controllable SLD strategy can further guide the development of novel CCAs with superior microstructure stability at elevated temperatures. It can be potentially applied to other metallic alloys.

## Methods

### Alloy fabrication

Four experimental alloy ingots were arc-melted with high-purity raw metals (Fe, Co, Cr, Ni, Ti, Al, and B, >99.9 % pure) under a Ti-getter argon atmosphere. The nominal compositions are respectively Ni_59.9-*x*_Co_*x*_Fe_13_Cr_15_Al_6_Ti_6_B_0.1_ (*x* = 0, 15, 30, and 60 at.%). For convenience, the alloys studied are referred to as the 0Co, 15Co, 30Co, and 60Co CCAs, respectively. Each ingot was re-melted at least five times to ensure the composition homogeneity, and then dropped into a copper mold with a size of 5 × 12 × 50 mm^3^. The as-cast samples were homogenized at 1165 °C for 2 h, followed by cold-rolling with a thickness reduction of ~65% along the longitude direction. The rolled samples were fully recrystallized at 1165 °C for ~2 min. Subsequently, isothermal aging treatments were carried out at 800, 900, and 1000 °C for various durations (24 h, 72 h, 168 h, and 240 h), and then cooled to room temperature in air.

### Microstructural characterizations

A SEM (FEI Scios) was employed to observe the microstructure features of the studied CCAs. The SEM samples were firstly ground mechanically using SiC papers and then electro-polished in a solution of C_2_H_5_OH and HNO_3_ (3:1) with a direct voltage of 20 V at −30 °C. A TEM (JEOL 2100 F) equipped with an EDS was applied to identify the crystal structure and chemical composition of different phases. The TEM samples were firstly ground to a thickness of 50 μm using SiC papers and then punched into discs with a diameter of 3 mm, followed by ion-milling to electron transparency via a precision ion polishing system (PIPS, Gatan 695). Regarding the quantitative evaluation of L1_2_ precipitates, the radius is evaluated to be *a*/2, where *a* is the average edge length of the cubic precipitates and the mean diameter of spherical precipitates. About 200 L1_2_ precipitates were measured for each aging condition. The APT experiment was performed using a local electrode atom probe (CAMECA, LEAP 5000 XR) in a voltage-pulsing mode at 70 K with a pulse rate of 200 kHz and a pulse fraction of 20%. The needle-shaped specimen was prepared using a FEI Scios dual-beam SEM/focused-ion-beam instrument. Image Visualization and Analysis Software package (IVAS 3.8.2) was used for quantitative compositional analysis and 3D reconstruction.

### Mechanical tests

Flat dog-bone-shaped tensile specimens with a gauge length of 12.5 mm, a width of 3.2 mm, and a thickness of ~1.5 mm were fabricated using electrical-discharge machining. Room temperature tensile properties were evaluated using a Material Testing System (MTS) tension machine with a strain rate of 1 × 10^−3 ^ s^−1^.

### Numerical computation of interdiffusion coefficient matrices

In this work, a free-accessible code HitDIC^52^ is employed to evaluate the interdiffusion coefficient matrices of FCC phase in the present multicomponent Ni-Co-Fe-Cr-Al-Ti systems. The pragmatic numerical inverse method^53^ is incorporated in the code, which can effectively evaluate the composition-dependent interdiffusion coefficients based on our experimental composition profiles (see Supplementary Tables. 1 and 2). Here, the interdiffusion coefficients of Al, Co, Cr, Fe, and Ti elements (with Ni as the solvent) were separately calculated in the three CCAs at 800, 900, and 1000 °C using the HitDIC software, as displayed in Fig. 4.

### First-principles calculations

First-principles calculations were performed using the Vienna ab initio simulation package based on density-functional theory (DFT)^54,55^. Ion-electron interactions were described by the projector augmented wave (PAW) approach^56^. The exchange-correlation interactions were modeled by the Perdew-Burke-Ernzerhof (PBE) form^57^. The Methfessel-Paxton smearing^58^ with a smearing width of 0.2 eV was employed. The cutoff energy of plane waves was 400 eV. The migration energy of a vacancy was calculated within a 4 × 4 × 4 FCC supercell containing 254 atoms, with a 2 × 2 × 2 k-point mesh. The energy of the saddle structure was calculated by directly optimizing the saddle configuration, with a force convergence criterion of 0.03 eV/Å^59^. To elucidate the effects of Co atoms in the local regions on the migration barriers, the 1NN shell of the vacancy and one of its nearest neighbors are assigned as Co atoms purposely, while all the rest atoms are randomly populated with equiatomic Co/Fe/Ni/Cr atoms. The ELF is proposed by Becke and Edgecombe to measure the probability of finding an electron pair in a space region. ELF provides a quantitative description of the electron distributions in the presence of other electrons. The ELF only takes values in the range between 0 and 1, where ELF = 1 corresponds to the perfect localization. In this work, the ELF distribution was calculated to quantify the bonding strength between different atoms based on the implementation in the VASP code^49^.

## Supplementary information

Supplementary Information

## Data Availability

The data that support the findings of this work are available from the corresponding authors upon request.

## Code availability

The codes used for the diffusion kinetic simulations and DFT calculations in this study are available from the corresponding authors upon request.
