# Supplementary material for: Achieving thermally stable nanoparticles in chemically complex alloys via controllable sluggish lattice diffusion
Source: Nat Commun. 2022 Aug 18;13:4870. doi: 10.1038/s41467-022-32620-6 (PMC9388539; doi:10.1038/s41467-022-32620-6)
Supplement: Supplementary file 1 — Supplementary Information [file 41467_2022_32620_MOESM1_ESM.pdf]

Supplementary Information

Achieving thermally stable nanoparticles in chemically complex alloys via controllable sluggish lattice diffusion

Bo Xiao[1,2,3], Junhua Luan[1], Shijun Zhao[2], Lijun Zhang[4], Shiyao Chen[4], Yilu Zhao[5], Lianyong Xu[6], C.T. Liu[1,3], Ji-Jung Kai[2,*], Tao Yang[1,3,*]

[1] Department of Materials Science and Engineering, City University of Hong Kong, Hong Kong, China;

[2] Department of Mechanical Engineering, City University of Hong Kong, Hong Kong, China;

[3] Hong Kong Institute for Advanced Study, City University of Hong Kong, Hong Kong, China;

[4] State Key Laboratory of Powder Metallurgy, Central South University, Changsha 410083, China;

[5] School of Materials Science and Engineering, Harbin Institute of Technology (Shenzhen), Shenzhen 518055, China;

[6] School of Materials Science and Engineering, Tianjin University, Tianjin 300350, China.

[*]Corresponding author: Ji-Jung Kai (jijkai@cityu.edu.hk), Tao Yang (taoyang6-c@my.cityu.edu.hk)

Supplementary Figures

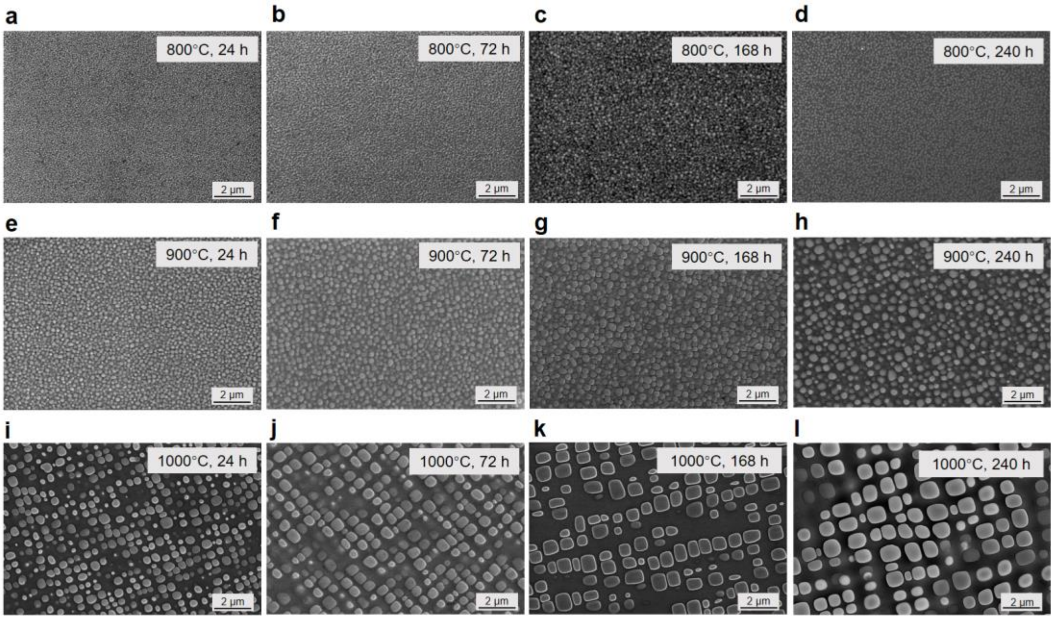

Supplementary Figure 1. Temporal evolution of nanoparticles in the 0Co CCA after thermal aging. a, 24 h/800 °C, **b,** 72 h/800 °C, **c,** 168 h/800 °C, **d,** 240 h/800 °C, **e,** 24 h/900 °C, **f,** 72 h/900 °C, **g,** 168 h/900 °C, **h,** 240 h/900 °C, **i,** 24 h/1000 °C, **j,** 72 h/1000 °C, **k,** 168 h/1000 °C, and **l,** 240 h/1000 °C.

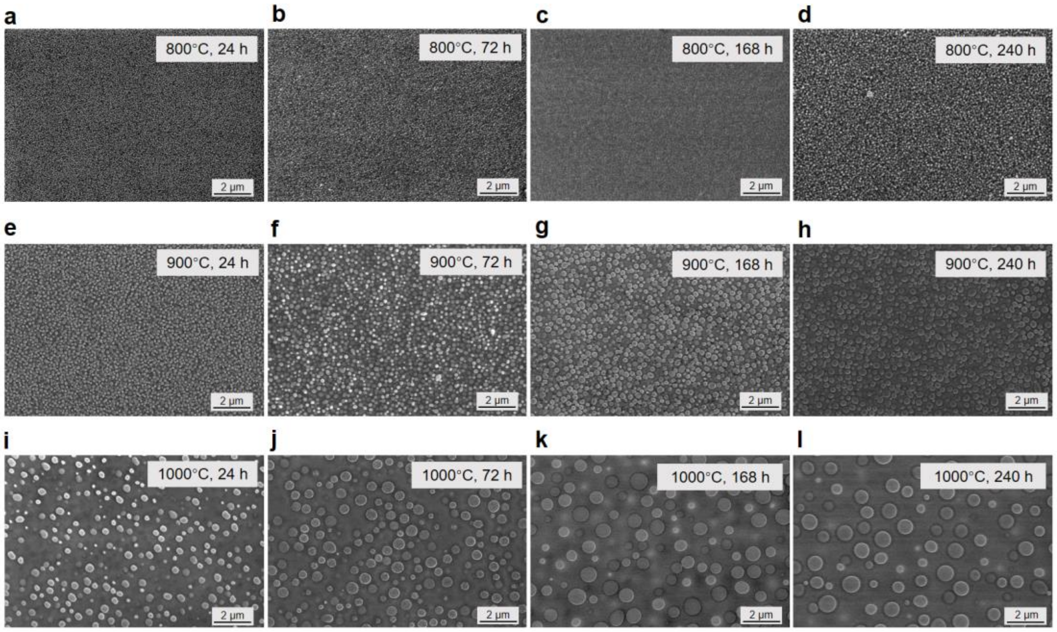

Supplementary Figure 2. Temporal evolution of nanoparticles in the 15Co CCA after thermal aging. a, 24 h/800 °C, **b,** 72 h/800 °C, **c,** 168 h/800 °C, **d,** 240 h/800 °C, **e,** 24 h/900 °C, **f,** 72 h/900 °C, **g,** 168 h/900 °C, **h,** 240 h/900 °C, **i,** 24 h/1000 °C, **j,** 72 h/1000 °C, **k,** 168 h/1000 °C, and **l,** 240 h/1000 °C.

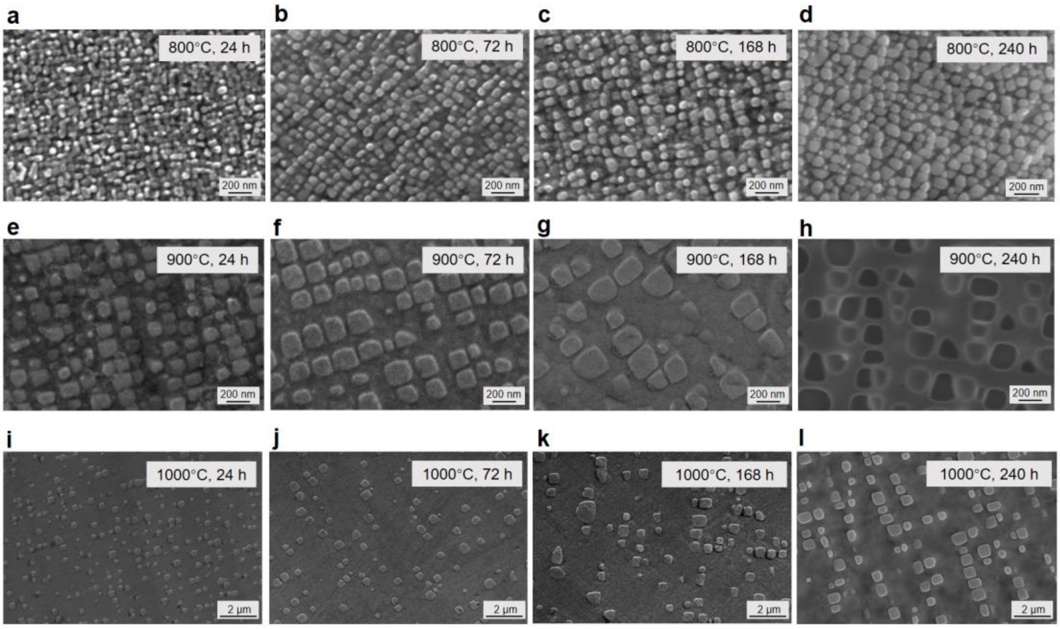

Supplementary Figure 3. Temporal evolution of nanoparticles in the 30Co CCA after thermal aging. a, 24 h/800 °C, **b,** 72 h/800 °C, **c,** 168 h/800 °C, **d,** 240 h/800 °C, **e,** 24 h/900 °C, **f,** 72 h/900 °C, **g,** 168 h/900 °C, **h,** 240 h/900 °C, **i,** 24 h/1000 °C, **j,** 72 h/1000 °C, **k,** 168 h/1000 °C, and **l,** 240 h/1000 °C.

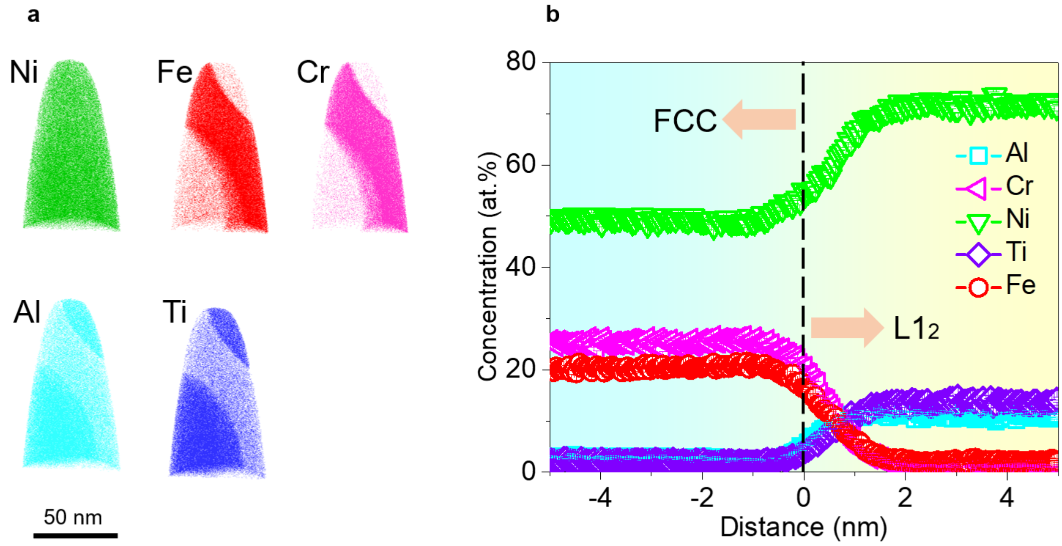

Supplementary Figure 4. The chemical composition of nanoparticles of the 0Co samples aged at 900 °C for 24 h. a, 3D-APT reconstruction of the tip, **b,** the corresponding compositional profile across the FCC/$L1_2$ interface.

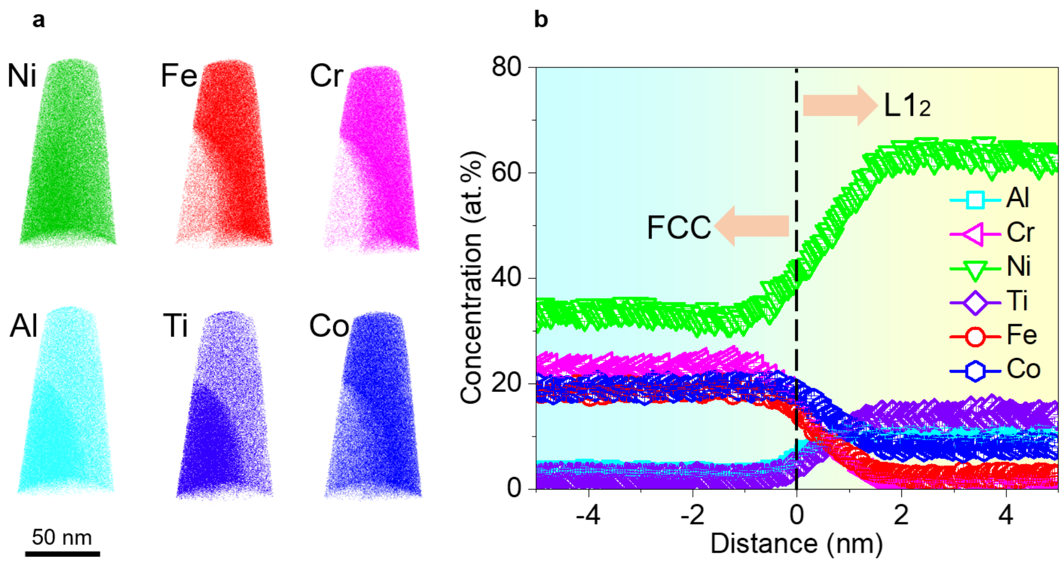

Supplementary Figure 5. The chemical composition of nanoparticles of the 15Co samples aged at 900 °C for 24 h. a, 3D-APT reconstruction of the tip, **b,** the corresponding compositional profile across the FCC/$L1_2$ interface.

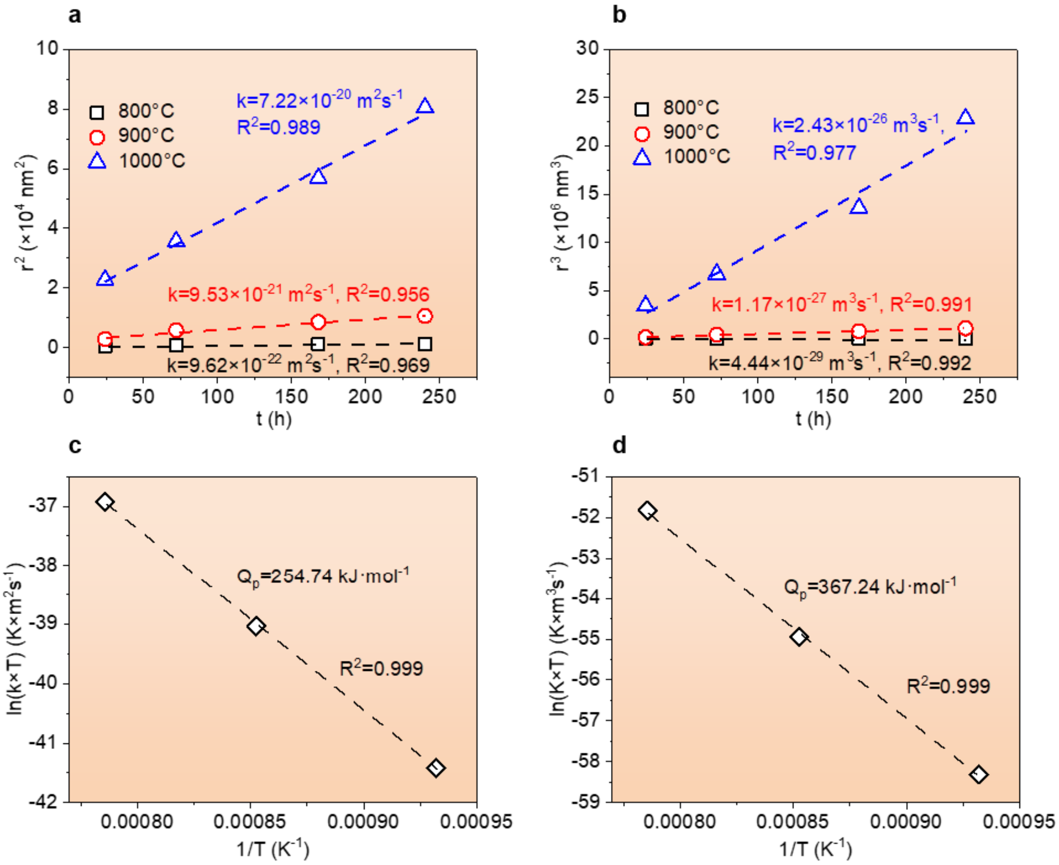

Supplementary Figure 6. Coarsening kinetics and associated mechanisms of $L1_2$ precipitates in the 30Co CCA. a, b, Plots of r^2 vs. t and r^3 vs. t of $L1_2$ precipitates in the 30Co CCA aged at 800, 900, and 1000 °C. **c, d,** Arrhenius plot of the coarsening rate constant (ln(k×T)) as a function of the reciprocal aging temperature (1/T) determines the activation energy (Q) for the 30Co CCA based on the TIDC and LSW model.

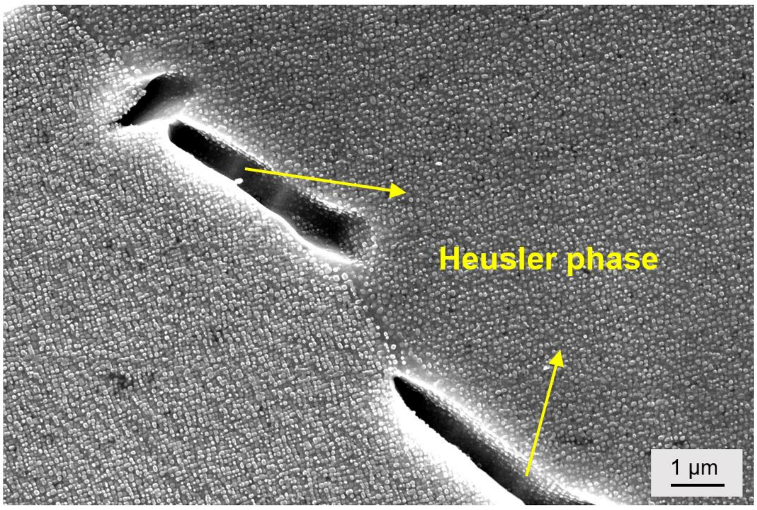

Supplementary Figure 7. SEM image showing the formation of Heusler phase in the 30Co CCA aged at 800 °C for 168 h. The Heusler phase starts to form along grain boundaries when the Co content increases up to 30 at.%, as indicated by yellow arrows.

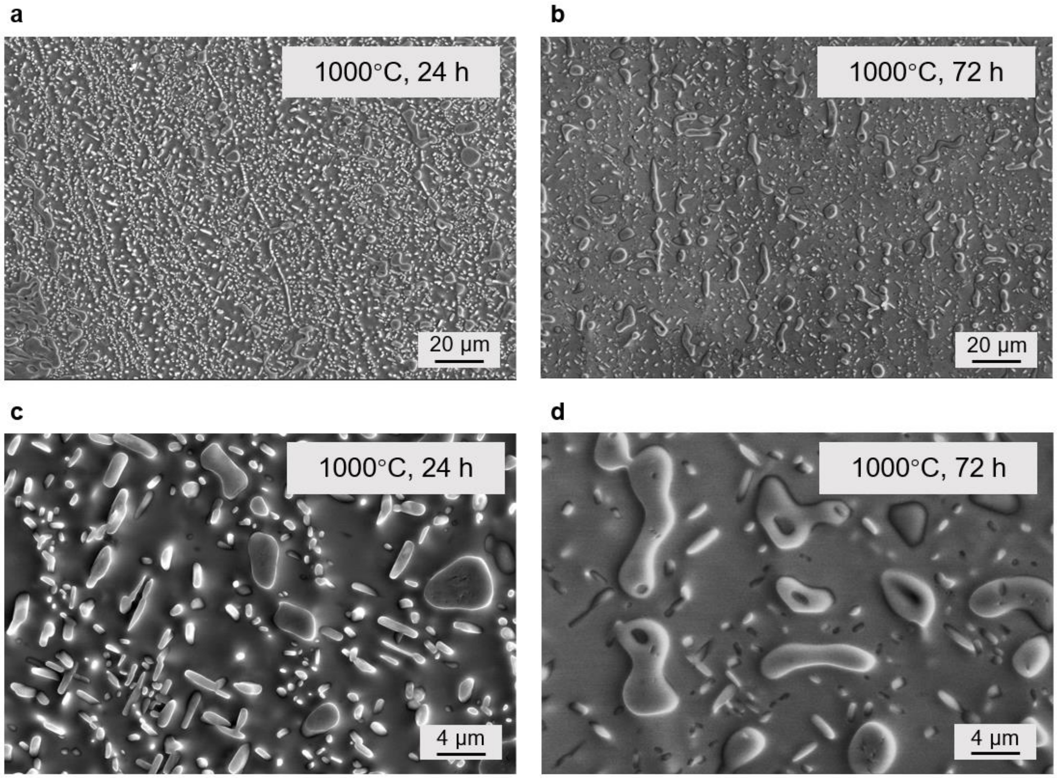

Supplementary Figure 8. SEM images of the 60Co CCA after thermal aging. a, 24 h/1000 °C, **b,** 72 h/1000 °C, **c, d,** magnified SEM images from the selected area in **a**, **b**.

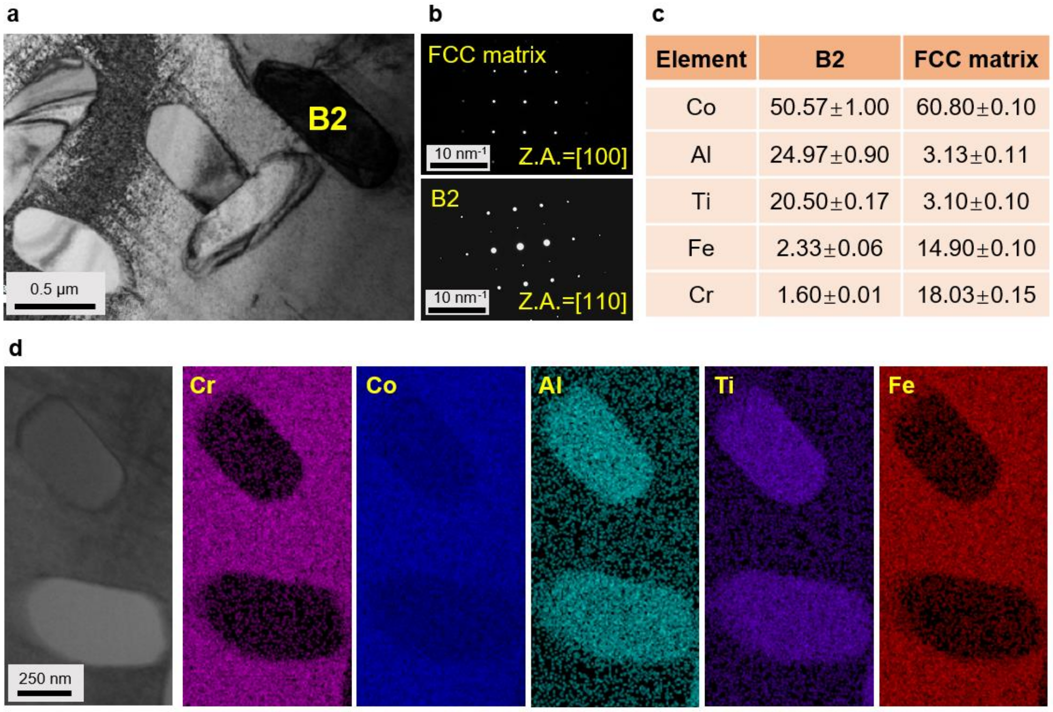

Element	B2	FCC matrix
Co	50.57±1.00	60.80±0.10
Al	24.97±0.90	3.13±0.11
Ti	20.50±0.17	3.10±0.10
Fe	2.33±0.06	14.90±0.10
Cr	1.60±0.01	18.03±0.15

Supplementary Figure 9. The crystal structure and chemical composition of particles and matrix for the 60Co CCA at 1000 °C for 24 h. a, bright-field TEM image showing elongated-like precipitates, **b,** FCC matrix and B2 precipitates with the SAED along [100] and [110] zone axes, **c,** chemical composition of B2 precipitates and FCC matrix, **d,** Scanning TEM EDS maps of the selected area for alloying elements of Cr, Co, Al, Ti, and Fe.

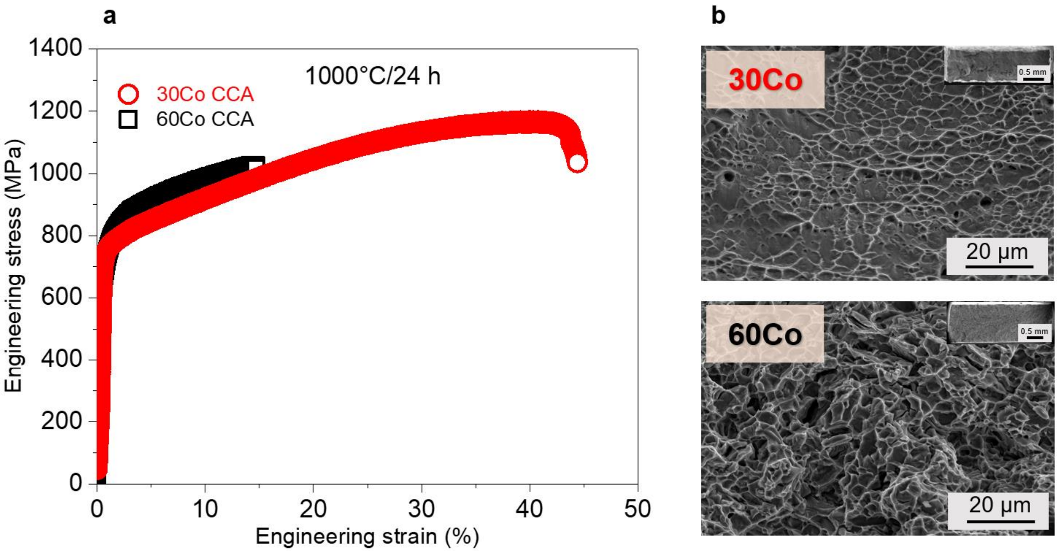

Supplementary Figure 10. Tensile properties and fracture morphologies of the 30Co and 60Co CCAs at ambient temperature. a, Engineering stress-strain curves indicate that the 60Co CCA shows an inferior tensile strength and ductility when compared to the 30Co CCA. **b**, The fracture morphologies reveal that the 30Co CCA exhibits ductile dimpled structures whereas the 60Co CCA shows the intergranular fracture mode.

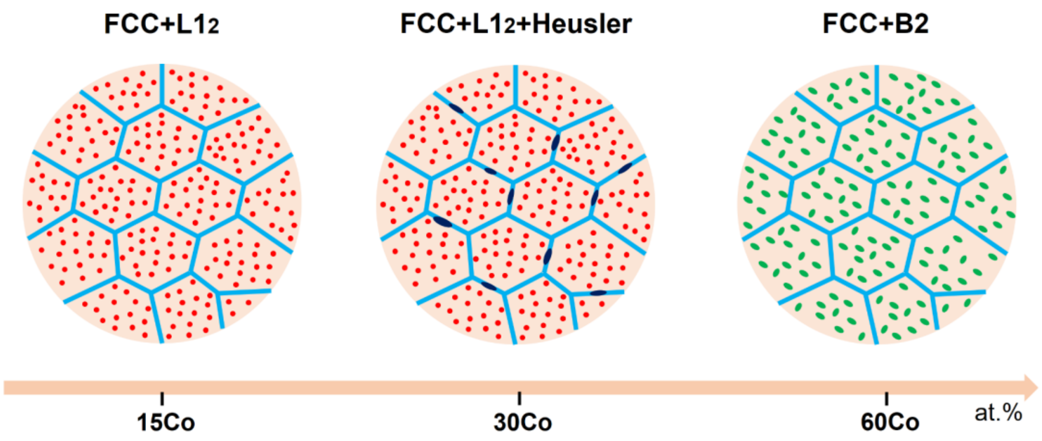

Supplementary Figure 11. Evolution of phase structures in the CCA system with increasing the Co content. The typical microstructure in the 15Co CCA consists of an FCC matrix with a high density of $L1_2$ precipitates. With increasing the Co content to 30 at.%, the brittle Heusler phase starts to precipitate at grain boundaries, accompanied with an FCC matrix and dense $L1_2$ precipitates. When the Co content further increases up to 60 at.%, it is composed of an FCC matrix with coarse B2 phase.

Supplementary Tables

Supplementary Table 1. Chemical compositions and partitioning of $L1_2$ precipitates and FCC matrix in the 0Co CCA aged at 800, 900, and 1000 °C for 24 h.

T	Phases	Chemical composition (at.%)					
		Ni	Co	Fe	Cr	Al	Ti
800	$L1_2$	72.23±0.50	—	2.08±0.20	1.75±0.19	11.20±0.26	12.74±0.34
	FCC	48.33±0.32	—	23.25±0.40	25.62±0.44	2.01±0.10	0.79±0.08
	K_i	1.49	—	0.09	0.07	5.57	16.13
900	$L1_2$	71.94±0.45	—	2.21±0.15	1.28±0.10	10.95±0.36	13.62±0.47
	FCC	49.42±0.29	—	20.17±0.23	25.38±0.38	2.82±0.08	2.21±0.09
	K_i	1.46	—	0.11	0.05	3.88	6.16
1000	$L1_2$	69.31±0.15	—	3.31±0.07	2.02±0.06	11.46±0.16	13.89±0.15
	FCC	53.68±0.30	—	17.40±0.25	21.23±0.38	4.68±0.20	3.01±0.19
	K_i	1.29	—	0.19	0.10	2.45	4.62

Supplementary Table 2. Chemical compositions and partitioning of $L1_2$ precipitates and FCC matrix in the 15Co CCA aged at 800, 900, and 1000 °C for 24 h.

T	Phases	Chemical composition (at.%)					
		Ni	Co	Fe	Cr	Al	Ti
800	$L1_2$	64.95±0.25	6.74±0.10	2.08±0.09	1.50±0.08	10.82±0.25	13.92±0.21
	FCC	28.82±0.16	20.62±0.18	20.07±0.19	25.83±0.13	2.51±0.08	2.16±0.05
	K_i	2.25	0.33	0.10	0.06	4.31	6.44
900	$L1_2$	63.74±0.73	8.10±0.43	2.60±0.20	1.59±0.15	10.16±0.38	13.81±0.39
	FCC	33.68±0.41	19.01±0.38	18.70±0.47	22.80±0.48	3.38±0.13	2.43±0.24
	K_i	1.89	0.43	0.14	0.07	3.01	5.68
1000	$L1_2$	60.45±0.36	8.64±0.20	3.40±0.22	2.15±0.21	10.87±0.44	14.49±0.11
	FCC	43.17±0.38	16.84±0.04	14.31±0.32	17.17±0.21	4.77±0.04	3.74±0.22
	K_i	1.40	0.51	0.24	0.13	2.28	3.88

Supplementary Table 3. The simulated interdiffusion coefficients of Al, Co, Cr, Fe, and Ti elements in FCC matrices (with Ni as the solvent) in the $Ni_{59.9-x}Co_xFe_{13}Cr_{15}Al_6Ti_6B_{0.1}$ (at.%) system at 800, 900, and 1000 °C.

Alloys	*T* (°C)	Interdiffusion coefficients of alloying elements (m/s)				
		Al	Co	Cr	Fe	Ti
0Co	800	1.06×10^{-14}	—	1.01×10^{-14}	9.13×10^{-15}	3.10×10^{-16}
	900	4.83×10^{-14}	—	2.57×10^{-14}	1.97×10^{-14}	2.16×10^{-15}
	1000	1.92×10^{-13}	—	3.16×10^{-14}	2.02×10^{-14}	3.49×10^{-15}
15Co	800	4.57×10^{-19}	4.61×10^{-19}	3.30×10^{-18}	9.25×10^{-19}	7.72×10^{-20}
	900	1.51×10^{-17}	8.58×10^{-18}	4.06×10^{-17}	1.58×10^{-17}	1.74×10^{-18}
	1000	3.45×10^{-16}	1.17×10^{-16}	3.59×10^{-16}	1.77×10^{-16}	3.94×10^{-17}
30Co	800	1.53×10^{-20}	3.19×10^{-19}	7.03×10^{-19}	1.27×10^{-18}	3.03×10^{-18}
	900	1.24×10^{-18}	6.92×10^{-18}	7.29×10^{-18}	2.62×10^{-17}	2.66×10^{-17}
	1000	3.41×10^{-17}	7.63×10^{-17}	7.91×10^{-17}	1.37×10^{-16}	2.13×10^{-16}

Supplementary Table 4. The evolution of the ELF values of the present CCA system with increasing the number of the nearest neighbor Co atoms.

Content	0Co	4Co	8Co
ELF value	0.39	0.43	0.44

Supplementary Note 1

Criterion for the transition of coarsening mechanisms of $L1_2$ precipitates

As is well-known, a high activation energy suggests a high diffusion barrier of vacancy formation and migration for the diffusion species, which significantly affects the coarsening behavior. In other words, the greater the activation energy, the slower the coarsening kinetics. Our experiments reveal that the addition of Co element reduces the coarsening kinetics of $L1_2$ precipitates, and suggests that the activation energy increases with increasing the Co content. According to **Fig. 3**, we assume that the coarsening mechanism of the 0Co CCA is controlled by TIDC mechanism, and its activation energy is therefore suggested to be 265.23 $kJ \cdot mol^{-1}$. Considering the coarsening behaviors with the Co addition, the activation energy of the 15Co CCA should be greater (probably 328.01 $kJ \cdot mol^{-1}$) when compared to the 0Co CCA (265.23 $kJ \cdot mol^{-1}$), and LSW mechanism is probably responsible for the 15Co CCA. Similarly, the 30Co CCA was also evaluated to be dominated by the LSW mechanism (367.24 $kJ \cdot mol^{-1}$, see **Supplementary Fig. 6**). For another case, we assume that the coarsening mechanism of the 0Co CCA is governed by the LSW mechanism, and its activation energy should be 380.57 $kJ \cdot mol^{-1}$. However, the calculated activation energies of the 15Co CCA by the TIDC and LSW mechanisms are respectively to be 222.02 $kJ \cdot mol^{-1}$ and 328.04 $kJ \cdot mol^{-1}$, less than 380.57 $kJ \cdot mol^{-1}$, both of which are not consistent with our experiments. Based on the above analysis, we suggest that the addition of Co element results in the transition of the coarsening mechanisms of $L1_2$ precipitates in such a CCA system, i.e., TIDC (0Co CCA) → LSW (15Co CCA) → LSW (30Co CCA).
